# Favorable liver and skeletal muscle changes in patients with MASLD and T2DM receiving glucagon-like peptide-1 receptor agonist: A prospective cohort study

**DOI:** 10.1097/MD.0000000000038444

**Published:** 2024-06-07

**Authors:** Tatsuya Kakegawa, Katsutoshi Sugimoto, Kazuhiro Saito, Daisuke Yunaiyama, Yoichi Araki, Takuya Wada, Hiroshi Takahashi, Yu Yoshimasu, Hirohito Takeuchi, Takao Itoi

**Affiliations:** a Department of Gastroenterology and Hepatology, Tokyo Medical University, Shinjuku-ku, Japan; b Department of Radiology, Tokyo Medical University, Shinjuku-ku, Japan.

**Keywords:** metabolic dysfunction-associated steatotic liver disease, semaglutide, skeletal muscle, type 2 diabetes mellitus

## Abstract

To investigate changes in skeletal muscle mass and fat fraction in patients with metabolic dysfunction-associated steatotic liver disease (MASLD) and type 2 diabetes mellitus (T2DM) undergoing treatment with Semaglutide for 6months. This single-arm pilot study included 21 patients with MASLD who received semaglutide for T2DM. Body weight, metabolic parameters, liver enzymes, fibrosis markers, skeletal muscle index (cm^2^/m^2^), and fat fraction (%) at the L3 level using the two-point Dixon method on magnetic resonance imaging (MRI), as well as liver steatosis and liver stiffness assessed using MRI-based proton density fat fraction (MRI-PDFF) and MR elastography, respectively, were prospectively examined before and 6 months after semaglutide administration. The mean age of the patients was 53 years and 47.6% were females. The median liver steatosis-fraction (%) and skeletal muscle steatosis-fraction values (%) significantly decreased (22.0 vs 12.0; *P* = .0014) and (12.8 vs 9.9; *P* = .0416) at baseline and 6 months, respectively, while maintaining muscle mass during treatment. Semaglutide also dramatically reduced hemoglobin A1c (%) (6.8 vs 5.8, *P* = .0003), AST (IU/L) (54 vs 26, *P* < .0001), ALT (IU/L) (80 vs 34, *P* = .0004), and γ-GTP (IU/L) levels (64 vs 34, *P* = .0007). Although not statistically significant, Body weight (kg) (79.9 vs 77.4), body mass index (BMI) (kg/m^2^) (28.9 vs 27.6), and liver stiffness (kPa) (28.9 vs 27.6) showed a decreasing trend. Fibrosis markers such as M2BPGi, type IV collagen, and skeletal muscle area did not differ. Semaglutide demonstrated favorable effects on liver and skeletal muscle steatosis, promoting improved liver function and diabetic status.

## 1. Introduction

Recent advances in the treatment of viral hepatitis have considerably improved its prognosis, allowing viral eradication or suppression in the majority of cases.^[1,2]^ Consequently, there has been a significant increase in the prevalence of nonalcoholic fatty liver disease (NAFLD) among individuals with chronic liver disease. NAFLD now affects over 25% of the global population.^[3]^ The clinical and histological spectrum of NAFLD ranges from nonalcoholic fatty liver (NAFL) to nonalcoholic steatohepatitis (NASH). Recently, the term “NAFLD” has been replaced with metabolic dysfunction-associated steatotic liver disease (MASLD) and “NASH” with “metabolic dysfunction-associated steatohepatitis” (MASH) due to its potentially stigmatizing language.^[4]^

Insulin resistance, a common feature of type 2 diabetes (T2DM) and obesity, is a key pathogenic driver of MASH.^[5]^ Glucagon-like peptide-1 receptor agonists (GLP-1RAs) induce weight loss and improve glycemic control in patients with obesity and T2DM. Although no pharmacotherapies have been approved for MASH treatment thus far, the biological effects of GLP-1RAs make them attractive agents for treating MASH.^[6,7]^

Currently, the medical interest is heavily focused on liver steatosis. Myosteatosis, characterized by intramuscular and intermuscular fat infiltration, is considered a significant pathological change in the process of sarcopenia, especially in the early phase, and is associated with insulin resistance and muscle dysfunction.^[8]^ It is common in patients with advanced age, diabetes, obesity, and malignant diseases.^[8]^ Therefore, developing effective prevention and treatment strategies is essential not only for enhancing patient care but also for promoting healthy aging.

In this study, we aimed to investigate changes in skeletal muscle mass and fat fraction, obesity severity, glucose and fat metabolism, and liver stiffness and fat fraction in patients with MASLD and T2DM undergoing treatment with the GLP-1RA, Semaglutide, for 6 months, to study the implications of GLP-1RAs treatment on myosteatosis and muscle mass in these patients.

## 2. Methods

### 2.1. Study design and patients

This was a single-center, open-label, single-arm clinical trial conducted over 24 weeks using semaglutide in patients with T2DM and MASLD. Participants were enrolled at Tokyo Medical University Hospital. The study was approved by the ethics committee of the hospital, and all treatments were provided as part of routine care for the participants. Each participant provided written informed consent prior to inclusion in the study.

Participants enrolled in this study were patients with MASLD who were receiving semaglutide treatment for the first time for T2DM and who had hemoglobin A1c (HbA1c) levels of ≥ 6.2% despite dietary/exercise therapies and/or the use of other antidiabetic drugs. MASLD was diagnosed using magnetic resonance imaging-based proton density fat fraction (MRI-PDFF), showing value ≥ 5.2%^[9]^ within 1 month prior to semaglutide therapy. Patients were excluded if they were under the age of 20 years, engaged in substantial alcohol consumption (>20 g/d for females or > 30 g/d for males), were diagnosed with Child–Pugh B/C cirrhosis, had other causes of liver disease, or had received new administration of vitamin E or antidiabetic drugs within 3 months before semaglutide treatment.

### 2.2. Treatment

The treatment with semaglutide was initiated at a dose of 0.25 mg once a week for the first week, and then increased to 0.5 mg once a week, to improve gastrointestinal tolerability. If the treatment effect was found to be inadequate, the dose was permitted to be increased to 1.0 mg once a week with continuous monitoring for adverse events using the Common Terminology Criteria for Adverse Events version 5.0.

### 2.3. Physical examination and serum biochemistry

Clinical and laboratory data were collected within 1 week before treatment and at the 6-month mark after treatment. Body mass index (BMI) was calculated as weight (kg) divided by height squared (m²). Laboratory data included aspartate aminotransferase (AST), alanine aminotransferase (ALT), gamma-glutamyl transpeptidase (-GTP), low-density lipoprotein cholesterol, high-density lipoprotein (HDL) cholesterol, triglycerides, HbA1c, type IV collagen 7s, and Mac-2 binding protein glycosylation isomer (M2BPGi) levels. Venous samples were collected from all participants after an overnight fast.

### 2.4. Liver stiffness measurement

All patients underwent liver MRI at 3 T (Skyra; Siemens Healthcare, Munich, Germany) using a 32-channel phased-array coil. Continuous longitudinal mechanical waves (60 Hz) were generated for MRE using a passive acoustic driver placed against the anterior chest wall. A two-dimensional spin-echo planar MRE sequence was employed to acquire axial wave images with the following parameters: repetition time/echo time = 1200 ms/47 ms; continuous sinusoidal vibration = 60 Hz; external driver amplitude = 50%; field of view = 400 mm; matrix size = 256 × 256; flip angle = 90°; section thickness = 5 mm; bandwidth = 2175 Hz/Px; 4 evenly spaced phase offsets; and 4 pairs of 60-Hz trapezoidal motion encoding gradients with zeroth and first-moment nulling along the through-plane direction. All processing steps were applied automatically without manual intervention, yielding quantitative images of tissue shear stiffness in kilopascals (kPa). On each section of the MRE images, regions of interests (ROIs) were plotted to include only the parenchyma of the right lobe while avoiding the edges of the liver and large blood vessels. Noted that the ROIs were not necessarily as large as possible but set where good wave-propagating area referencing the wave images.^[10]^ The mean of the measurements from the 4 slices was used. This study was considered invalid if the liver parenchyma could not be measured using elastography with reliability maps. The regions of interest did not include regions in which the phase signal-to-noise ratio (the wave amplitude ratio to noise in the wave images) was less than 5 according to the Rose criterion.^[11]^

### 2.5. Liver steatosis measurement

The MRI-PDFF data was obtained using the multi-echo Dixon method with the following parameters: repetition time: 9 ms; echo time: 1.05, 2.46, 3.69, 4.92, 6.15, 7.38 ms; flip angle: 4°; matrix size of 320 × 280; axial imaging plane; section thickness of 6 mm; field of view (FOV) of 450 mm; fractional phase FOV ranging from 0.75 to 1; one signal acquired; bandwidth of 1080 Hz/Px; and imaging time consisting of 2 breath holds (approximately 16 seconds each). A single ROI (20 × 20 × 20 mm^3^) was placed in liver segment VII or VIII in a similar approach to the previous study.^[9]^ LSM and PDFF were analyzed by a radiologist with 27 years of experience who was blinded to the patients’ clinical information.

### 2.6. Skeletal muscle cross-sectional areas measurement

Skeletal muscle (SM) cross-sectional areas were quantified at the L3 vertebral level using the fat image obtained by the two-point Dixon method with the following parameters: repetition time of 4.2 ms; echo times of 1.34 and 2.57 ms; flip angle of 4°; matrix size of 320 × 240; axial imaging plane; section thickness, 6 mm; FOV, 450 mm; and fractional phase FOV ranging from 0.75 to 1; one signal acquired; and bandwidth of 1040 Hz/Px. The psoas, paraspinal, and abdominal wall muscles were semiautomatically delineated using SYNAPSE VINCENT Ver. 6.7 (FUJIFILM, Tokyo, Japan) by setting the cutoff number for the signal intensity. A radiologist with 15 years of experience and a radiological technologist with 27 years of experience determined the cutoff point by calculating the average maximum intensity of the ROI with an area of at least 100 mm^2^ on the bilateral psoas muscle. They obtained SM cross-sectional areas smaller than the cutoff point. The cross-sectional area (cm^2^) of the SM was adjusted for body size by dividing it by the square of height (m^2^) to generate the skeletal muscle index (SMI).

### 2.7. Skeletal muscle steatosis fraction measurement

Two signal intensity (SI) maps, SI_fat_ and SI_water_, were obtained using the two-point Dixon method on MR images because unlike liver steatosis muscle steatosis infiltrated heterogeneously into the skeletal muscle. The ROI of the two-point Dixon method was the same slice and area used for the SM cross-sectional area measurement. A 27-year experienced radiological technologist set the ROIs using the semi-automated contour method installed on SYNAPSE VINCENT Ver. 6.7 (FUJIFILM) on SI_fat_ images and copied the ROIs onto the SI_water_ images. The SM steatosis fractions were calculated using the following formula^[12]^:

SM steatosis fraction = 100 × SI_fat_/(SI_fat_ + SI_water_)

### 2.8. Clinical outcomes

The primary outcomes assessed were changes in both SM steatosis fractions and SMI between pre- and post-treatment statuses. Secondary clinical outcomes included factors associated with SM and liver steatosis fractions before treatment as well as changes in BMI, AST, ALT,-GTP, low-density lipoprotein-cholesterol, HDL-cholesterol, triglycerides, HbA1c, Type IV collagen 7s, M2BPGi, liver stiffness, and liver steatosis fraction before and after treatment.

### 2.9. Statistical approach

Continuous variables are reported as medians with interquartile ranges and compared using the Wilcoxon signed-rank test. Categorical variables are reported as percentages and compared using the χ^2^ test. Linear regression was performed to determine the predictors of pretreatment SM and liver steatosis fractions. Multivariable models were constructed using a backward elimination approach with pre-specified elimination criteria based on a significance threshold of 0.05. All statistical analyses were carried out with EZR version 1.55 (Saitama Medical Center, Jichi Medical University, Saitama, Japan) or GraphPad Prism version 9.0.1, and *P* values of < .05 were considered statistically significant.

## 3. Results

### 3.1. Baseline characteristics of the patients

Between July 17, 2020, and April 8, 2022, 23 patients were enrolled in this study. Two patients were lost to follow-up. Thus, 21 patients were included in the final analysis. Patient data before semaglutide treatment are shown in Table 1. There were 11 males and 10 females with a median age of 52 years (interquartile ranges, 49–64) years. The median BMI value was 28.9 (26.3–34.8) kg/m^2^. The median AST, ALT, and -GTP levels were 54 (40–78) U/L, 80 (48–116) U/L, and 64 (51–96) U/L, respectively. The median HbA1c was 6.9% (6.3–7.4%). Prior to semaglutide treatment, 18 (85.7%) received dietary and/or exercise therapies without any antidiabetic or antidyslipidemic drugs, whereas the remaining 6 (28.6%) received other antidiabetic drugs, including biguanides (n = 2), SGLT2-Is (n = 4), and insulin (n = 2). Four patients (19.0%) received anti-dyslipidemic drugs, including a selective PPARα modulator (n = 4) and statins (n = 1). The median values of the fibrosis markers were as follows:5.1 ng/mL (3.8–5.9) for Type IV collagen 7s and 0.62 C.O.I. (0.41–0.76) for M2BPGi. The median liver stiffness and liver steatosis-fraction were 3.01 kPa (2.62–4.75) and 22.0% (13.5–26.0), respectively. The median skeletal muscle steatosis-fraction and skeletal muscle cross-section area/hight^2^ were 12.8% (10.1–15.9) and 32.2 cm^2^/m^2^ (25.7–34.8), respectively.

**Table 1:** Baseline characteristics of the patients with nonalcoholic fatty liver disease and type 2 diabetes.

Variables	n = 21
Age (yr)	52 (49–64)
Sex (male/female)	11 (52.4)/10 (47.6)
Body weight (kg)	79.9 (68.4–88.1)
Body mass index (kg/m^2^)	28.9 (26.3–34.8)
AST (U/L)	54 (40–78)
ALT (U/L)	80 (48–116)
γ-GTP (U/L)	64 (51–96)
LDL-cholesterol (mg/dL)	116 (94–150)
HDL-cholesterol (mg/dL)	45 (41–55)
Triglyceride (mg/dL)	163 (112–219)
HbA1c (%)	6.8 (6.3–7.4)
Concomitant diabetes treatment of semaglutide	
No antidiabetic drugs	17 (81.0)
Biguanides	2 (9.5)
SGLTs-Is	4 (19.0)
Insulin	2 (9.5)
Anti-dyslipidemic drugs	4 (19)
Statins	1 (4.8)
Type IV collagen 7s (ng/mL)	5.1 (3.8–5.9)
M2BPGi (C.O.I.)	0.62 (0.41–0.76)
Liver stiffness (kPa)	3.01 (2.62–4.75)
Liver steatosis-fraction (%)	22.0 (13.5–26.0)
Skeletal muscle steatosis-fraction (%)	12.8 (10.1–15.9)
Skeletal muscle index (cm^2^/m^2^)	18.58 (16.01–20.74)

Data represent the numbers of patient observations with percentages in parentheses. Data in parentheses are the interquartile ranges.

ALT = alanine aminotransferase, AST = aspartate aminotransferase, HbA1c = hemoglobin A1c, HDL-cholesterol = high-density lipoprotein cholesterol, LDL-cholesterol = low-density lipoprotein cholesterol, M2BPGi = mac-2 binding protein glycosylated isomers, SGLTs-Is = sodium glucose cotransporter 2 inhibitors, γ-GTP = gamma-glutamyl transpeptidase.

### 3.2. Clinical parameters affecting pretreatment skeletal muscle steatosis-fraction and liver steatosis-fraction

In univariable linear regression analysis, age (β coefficient: 2.10 × 10^3^, 95% CI = 3.93 × 10^4^:3.78 × 10^3^, *P* = .0184), Liver stiffness (β coefficient: 2.17 × 10^2^, 95% CI = 2.07 × 10^3^:4.13 × 10^2^, *P* = .0320), and HDL-cholesterol (β coefficient: 8.78 × 10^4^, 95% CI = 4.24 × 10^5^:1.71 × 10^3^, *P* = .0405) were significantly associated with pretreatment skeletal muscle steatosis-fraction (Table 2). In multivariable linear regression, age (β coefficient: 1.68 × 10^3^, 95% CI = 2.03 × 10^4^:3.15 × 10^3^, *P* = .0281) and Liver stiffness (β coefficient: 1.70 × 10^2^, 95% CI = 2.03 × 10^4^:3.15 × 10^3^, *P* = .0447) were significantly associated with pretreatment skeletal muscle steatosis-fraction (Table 2). No significant clinical factors were associated with liver steatosis.

**Table 2:** Clinical factors associated with Skeletal muscle fat-fraction in baseline patients-linear regression.

Variable	β (95% CI)	*P* value	β (95% CI)	*P* value
Age	2.10 × 10^3^ (3.93 × 10^4^:3.78 × 10^3^)	.0184	1.68 × 10^3^ (2.03 × 10^4^:3.15 × 10^3^)	.0281
Body weight (kg)	−3.60 × 10^4^ (−1.91 × 10^3^:1.19 × 10^3^)	n.s.		
Body mass index (kg/m^2^)	1.30 × 10^3^ (−4.29 × 10^3^:6.89 × 10^3^)	n.s.		
AST (U/L)	3.31 × 10^4^ (−6.78 × 10^4^:1.34 × 10^3^)	n.s.		
ALT (U/L)	−8.11 × 10^5^ (−6.08 × 10^4^:4.45 × 10^4^)	n.s.		
γ-GTP (U/L)	2.95 × 10^4^ (−3.40 × 10^4^:9.29 × 10^4^)	n.s.		
LDL-cholesterol (mg/dL)	−7.29 × 10^4^ (−1.48 × 10^3^:1.93 × 10^5^)	n.s.		
HDL-cholesterol (mg/dL)	8.78 × 10^4^ (4.24 × 10^5^:1.71 × 10^3^)	.0405	6.34 × 10^4^ (−6.93 × 10^5^:1.34 × 10^3^)	n.s.
Triglyceride (mg/dL)	3.74 × 10^5^ (−2.07 × 10^4^:2.82 × 10^4^)	n.s.		
HbA1c (%)	1.60 × 10^2^ (−5.23 × 10^3^:3.72 × 10^2^)	n.s.		
Type IV collagen 7s (ng/mL)	8.75 × 10^3^ (−5.35 × 10^3^:2.28 × 10^2^)	n.s.		
M2BPGi (C.O.I.)	8.29 × 10^2^ (−3.31 × 10^3^:1.69 × 10)	n.s.		
Liver stiffness (kPa)	2.17 × 10^2^ (2.07 × 10^3^:4.13 × 10^2^)	.0320	1.70 × 10^2^ (2.03 × 10^4^:3.15 × 10^3^)	.0447
Liver fat-fraction (%)	−2.31 × 10^3^ (−6.48 × 10^3^:1.86 × 10^3^)	n.s.		
Skeletal muscle index (cm^2^/m^2^)	−2.22 × 10^5^ (−5.87 × 10^5^:1.44 × 10^5^)	n.s.		

ALT = alanine aminotransferase, AST = aspartate aminotransferase, CI = confidence interval, HbA1c = hemoglobin A1c, HDL-cholesterol = high-density lipoprotein cholesterol, LDL-cholesterol = low-density lipoprotein cholesterol, M2BPGi = mac-2 binding protein glycosylated isomers, n.s. = not significant, SGLTs-Is = sodium glucose cotransporter 2 inhibitors, γ-GTP = gamma-glutamyl transpeptidase.

### 3.3. Efficacy of semaglutide

**Figure 1:**
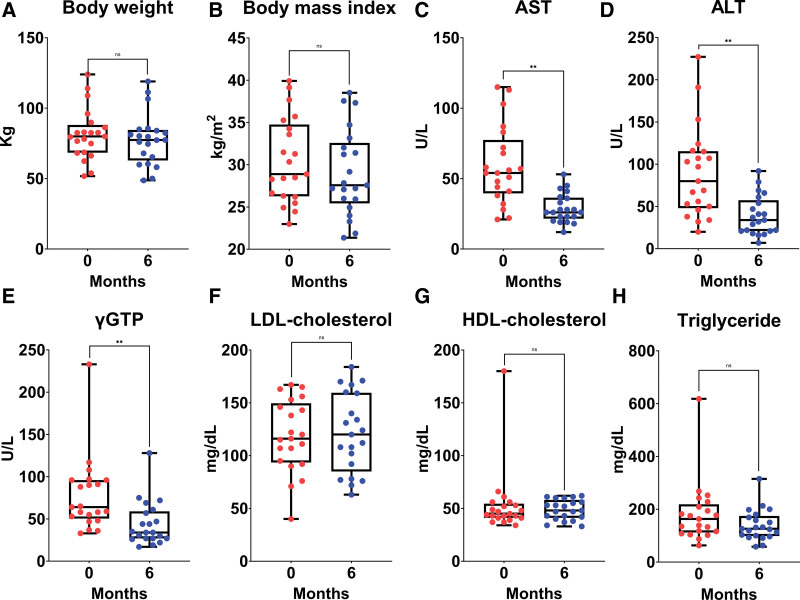
Box plots shows changes from baseline in (A) body weight, (B) body mass index, (C) aspartate aminotransferase (AST), (D) alanine aminotransferase (ALT), (E) gamma glutamyl transpeptidase (γ-GTP), (F) low-density lipoprotein cholesterol (LDL-cholesterol), (G) high-density lipoprotein cholesterol (HDL-cholesterol), and (H) triglyceride in patients treated with semaglutide for 6 months. *<.05, **<.01. ALT = alanine aminotransferase, AST = aspartate aminotransferase, HDL-cholesterol = high-density lipoprotein cholesterol, LDL-cholesterol = low-density lipoprotein cholesterol, n.s. = not significant, γ-GTP = gamma glutamyl transpeptidase.

**Figure 2.**
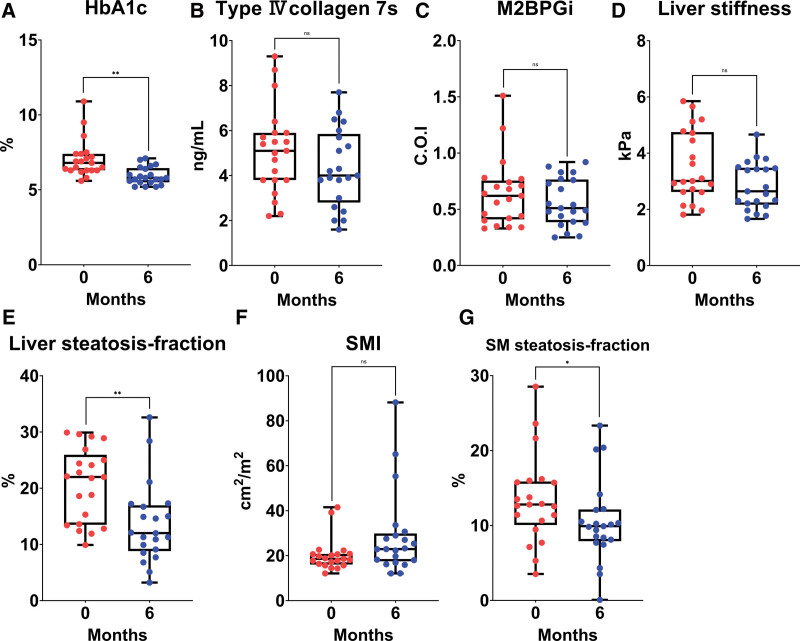
Box plots shows changes from baseline in (A) HbA1c, (B) Type IV collagen 7s, (C) M2BPGi, (D) Liver stiffness, (E) Liver steatosis-fraction, (F) Skeletal muscle steatosis-fraction, and (G) Skeletal muscle index in patients treated with semaglutide for 6 months. *<.05, **<.01. HbA1c = hemoglobin A1c, M2BPGi = mac-2 binding protein glycosylated isomers, n.s. = not significant.

**Figure 3:**
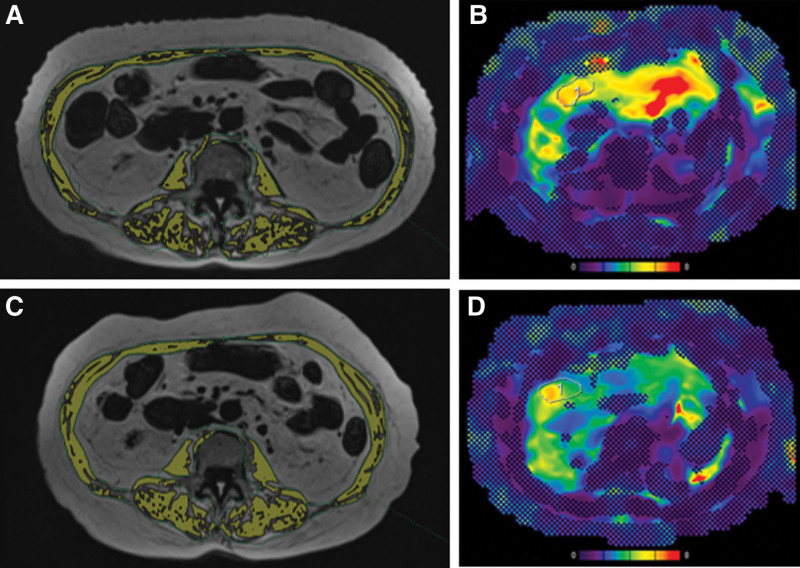
An 80-year-old woman diagnosed with MASLD and T2DM underwent treatment with semaglutide for a duration of 6 months. (A) pretreatment MR imaging at the L3 vertebral level illustrates the skeletal muscle (SM) cross-sectional areas (highlighted as yellow areas: 26.51 cm^2^) and SM steatosis areas (observed as a defect in yellow areas), with a calculated SM steatosis fraction of 28.5%. (B) pretreatment MRE and MRI-PDFF (image not shown) reveal liver stiffness measuring 5.66 kPa and a liver fat fraction of 12.8%. (C) Post-treatment MR imaging at the L3 vertebral level displays the SM cross-sectional areas (yellow areas: 26.49 cm²) and SM steatosis areas (identified as a defect in yellow areas), with a recalculated SM steatosis fraction of 23.3%. (D) Post-treatment MRE and MRI-PDFF assessments (image not shown) indicate a marked reduction in liver stiffness to 3.42 kPa and liver fat fraction to 3.2%. MASLD = metabolic dysfunction-associated steatotic liver disease, MR = magnetic resonance, MRE = magnetic resonance elastography, MRI-PDFF = magnetic resonance imaging-based proton density fat fraction, T2DM = type 2 diabetes mellitus.

The changes in each parameter from baseline to 6 months of semaglutide treatment are shown in Table 3, Figures 1 and 2. The median AST, ALT, and-GTP levels decreased significantly from 54 U/L (40–78), 80 U/L (48–116), and 64 U/L (51–96) at baseline to 26 U/L (22–37), 34 U/L (21–58), and 34 U/L (27–59) at 6 months, respectively. The median HbA1c value significantly decreased from 6.8% (6.3–7.4) at baseline to 5.8% (5.5–6.5) at 6 months. The median liver steatosis-fraction and skeletal muscle steatosis-fraction values significantly decreased from 22.0% (13.5–26.0) and 12.8% (10.1–15.9) at baseline to 12.0% (8.8–17.0) and 10.1% (7.9–12.1) at 6 months, respectively (Fig. 3).

**Table 3:** Changes in clinical characteristics in the patients who received semaglutide for 6 months.

Variables	Semaglutide therapy		
	Baseline (n = 21)	6 months (n = 21)	*P* value
Body weight (kg)	79.9 (68.4–88.1)	77.4 (62.9–84.5)	n.s.
Body mass index (kg/m^2^)	28.9 (26.3–34.8)	27.6 (25.4–32.6)	n.s.
AST (U/L)	54 (40–78)	26 (22–37)	<.0001
ALT (U/L)	80 (48–116)	34 (21–58)	.0004
γ-GTP (U/L)	64 (51–96)	34 (27–59)	.0007
LDL-cholesterol (mg/dL)	116 (94–150)	120 (85–160)	n.s.
HDL-cholesterol (mg/dL)	45 (41–55)	48 (41–58)	n.s.
Triglyceride (mg/dL)	163 (112–219)	126 (99–175)	n.s.
HbA1c (%)	6.8 (6.3–7.4)	5.8 (5.5–6.5)	.0003
Type IV collagen 7s (ng/mL)	5.1 (3.8–5.9)	4.0 (2.8–5.9)	n.s.
M2BPGi (C.O.I.)	0.62 (0.41–0.76)	0.51 (0.39–0.77)	n.s.
Liver stiffness (kPa)	3.01 (2.62–4.75)	2.64 (2.17–3.48)	n.s.
Liver steatosis-fraction (%)	22.0 (13.5–26.0)	12.0 (8.8–17.0)	.0014
Skeletal muscle steatosis-fraction (%)	12.8 (10.1–15.9)	9.9 (7.9–12.1)	.0416
Skeletal muscle index (cm^2^/m^2^)	18.58 (16.01–20.74)	22.89 (17.45–29.82)	n.s.

ALT = alanine aminotransferase, AST = aspartate aminotransferase, HbA1c = hemoglobin A1c, HDL-cholesterol = high-density lipoprotein cholesterol, LDL-cholesterol = low-density lipoprotein cholesterol, M2BPGi = mac-2 binding protein glycosylated isomer, n.s. = not significant, γ-GTP = gamma-glutamyl transpeptidase.

### 3.4. Adverse events

Among the adverse events, all were gastrointestinal disorders of grade 1 (mild) severity and were transient. Three patients (14.3%) had dyspepsia and 2 patients (9.5%) had grade I constipation. None of the patients prematurely discontinued treatment due to semaglutide-related adverse events.

## 4. Discussion

Our study demonstrated an improvement in body composition after 6 months of semaglutide therapy. Particularly, the degree of muscle steatosis (myosteatosis) significantly improved after 6 months of semaglutide therapy. Moreover, in addition to fat loss, no significant reduction in skeletal muscle mass was observed; instead, an upward trend was observed. Generally, loss of body fat mass and skeletal muscle mass occurs after caloric restriction and bariatric surgery.^[13]^ However, the results of studies on the effects of GLP-1RAs on lean body mass and skeletal muscle mass are controversial. In the LEAD-3 trial, liraglutide decreased body weight after treatment and induced the loss of lean body mass.^[14]^ In contrast, other studies have reported no change or increase in lean body mass.^[15,16]^ Sarcopenia and obesity can independently increase the risk of adverse health outcomes. Synergistically, these conditions can amplify health risks.^[17]^ Therefore, maintaining muscle mass during weight loss treatment is particularly important, as the preservation of skeletal muscle is the key to maintaining insulin sensitivity. Therefore, semaglutide might be an ideal treatment option for patients with myosteatosis.

Several studies have investigated the effects of GLP-1-based drugs on muscle mass, metabolism, and function. In an animal study, Ren et al^[18]^ studied the effects of semaglutide on skeletal muscles and its metabolomics. They concluded that semaglutide significantly reduced body weight and intramuscular fat accumulation and improved muscle function in obese mice by altering muscle lipid and organic acid metabolism. Hong et al^[19]^ also investigated the therapeutic potential of GLP-1RAs for muscle wasting and the mechanisms involved and concluded that GLP-1RAs ameliorated muscle wasting by modulating the relevant signaling pathways. Thus, GLP-1RAs may be useful for the treatment of myosteatosis and sarcopenic obesity.

Moreover, we demonstrated that the degree of muscle steatosis (myosteatosis) significantly increased in correlation with aging and liver stiffness, as measured using MRE. Previous studies have reported that myosteatosis is likely to occur in patients with Child–Turcotte–Pugh class C cirrhosis, and is associated with hepatic encephalopathy.^[20]^ Although all the participants in our study had Child–Turcotte–Pugh class A disease, myosteatosis increased in accordance with liver stiffness. As liver stiffness measured by MRE is closely related to the stage of liver fibrosis, our study suggests that myosteatosis is initiated at a relatively early stage of chronic liver disease. Furthermore, within our study cohort, 3 out of 21 patients (14.3%) showed no improvement in myosteatosis. Nonetheless, there were no significant differences in the serological and physical examination findings among them.

In this study, liver steatosis significantly improved after 6 months of semaglutide therapy. Although the fibrosis stage is a strong predictor of outcomes in patients with MASLD,^[21]^ the degree of hepatic fat accumulation has recently been hypothesized to play an essential role in NASH and fibrosis development.^[22]^ Ajmera et al^[23]^ showed that a high baseline liver fat percentage was associated with a substantial risk of subsequent fibrosis development in patients with MASLD without baseline fibrosis. Thus, loss of liver steatosis is vital not only for improving insulin sensitivity and preventing cardiovascular disease, but also for halting the progression of liver fibrosis, thereby preventing cirrhosis and hepatocellular carcinoma. In contrast, our findings showed no association between the degree of liver steatosis and clinical factors. Furthermore, regarding liver steatosis, within our study’s cohort, 2 out of 21 patients (9.5%) exhibited no improvement in liver steatosis. These two individuals had severe T2DM, with an average HbA1c of 10.2%, and were undergoing treatment with insulin and sodium-glucose cotransporter 2 inhibitors. We hypothesize that adequate glycemic control is critical for the amelioration of MAFLD in patients with T2DM.

Regarding liver fibrosis, which is a strong predictor of outcomes in patients with MASLD^[21]^ no consensus has been reached concerning the influence of GLP-1RAs on liver fibrosis in patients with MASLD. Placebo-controlled trials of subcutaneous liraglutide^[6]^ and semaglutide^[7]^ have shown that GLP-1RAs may inhibit the exacerbation of liver fibrosis, but do not result in significant improvement. Thus, the resolution of liver fibrosis may be delayed. Thus, long-term reduction of liver steatosis and consequent improvement in liver inflammation might lead to improved liver fibrosis. In this study, although not significant, liver stiffness measured by MRE and serum fibrosis markers, such as Type IV collagen 7s and M2BPGi, decreased after 6 months of semaglutide treatment. This phenomenon does not represent the resolution of liver fibrosis in 6 months, but rather a decrease in liver steatosis and improvement in liver inflammation. In this study, liver steatosis measured by MRI-PDFF and serum liver enzymes, such as AST, ALT, and-GTP, representing liver inflammation; significantly decreased after 6 months of semaglutide therapy.

Our study had several limitations. First, it was a single-arm, open-label study. Thus, there might be the effect of significant potential confounding factors, such as calorie intake and physical activity. Second, the sample size was small, albeit statistically adequate. Third, serial liver biopsy was not performed to assess changes in liver fibrosis stage and steatosis grade because the study was conducted within routine clinical practice. Although MRE is considered the most accurate noninvasive imaging-based biomarker of fibrosis in MASLD,^[9]^ the liver stiffness value may also be influenced by factors such as passive congestion, marked inflammation, and infiltrative diseases. Despite its drawbacks, liver biopsy remains the best method for providing detailed information on liver structure.^[19]^ Regarding the assessment of liver steatosis, MRI-PDFF is a proven optimal imaging biomarker which is routinely used in clinical research.^[9]^ Finally, our cohort predominantly comprised younger patients (median age: 52) and those with obesity (median BMI: 28.9 kg/m^2^). Consequently, additional research is required to ascertain if our findings are applicable to MAFLD patients with T2DM who are non-obese or of an older age group.

In conclusion, 6 months of semaglutide treatment demonstrated a significant reduction in both liver and skeletal muscle steatosis while maintaining skeletal muscle volume, leading to improved liver function and diabetic status in patients with MASLD complicated with T2DM. Semaglutide should be considered as an optimal approach for treating myosteatosis and liver steatosis in these patients. Further long-term studies with more extensive cohorts are required to confirm these findings.

## Acknowledgments

The authors would like to thank ahh of the patients and thir families as well as the staff of the participating institutions.

## Author contributions

**Conceptualization:** Tatsuya Kakegawa, Katsutoshi Sugimoto, Kazuhiro Saito, Daisuke Yunaiyama, Yoichi Araki, Takuya Wada, Hiroshi Takahashi, Yu Yoshimasu, Hirohito Takeuchi, Takao Itoi.

**Data curation:** Tatsuya Kakegawa, Katsutoshi Sugimoto, Kazuhiro Saito, Daisuke Yunaiyama, Yoichi Araki, Takuya Wada, Hiroshi Takahashi, Yu Yoshimasu, Hirohito Takeuchi, Takao Itoi.

**Formal analysis:** Tatsuya Kakegawa, Katsutoshi Sugimoto, Kazuhiro Saito, Daisuke Yunaiyama, Yoichi Araki, Takuya Wada, Hiroshi Takahashi, Yu Yoshimasu, Hirohito Takeuchi, Takao Itoi.

**Funding acquisition:** Takao Itoi.

**Investigation:** Katsutoshi Sugimoto, Daisuke Yunaiyama, Takuya Wada, Hiroshi Takahashi, Hirohito Takeuchi, Takao Itoi.

**Project administration:** Tatsuya Kakegawa, Katsutoshi Sugimoto.

**Writing – original draft:** Katsutoshi Sugimoto.
